# First trimester growth after fresh or frozen single embryo transfer: embryo cleavage vs blastocyst stages

**DOI:** 10.1093/hropen/hoag014

**Published:** 2026-02-17

**Authors:** Mujin Ye, Mariana Tavares Barroso, Anna Sara Oberg, Nermin Hadziosmanovic, Arturo Reyes Palomares, Kenny A Rodriguez-Wallberg

**Affiliations:** Department of Oncology-Pathology, Laboratory of Translational Fertility Preservation, Karolinska Institutet, Stockholm, Sweden; Department of Medical Epidemiology and Biostatistics, Karolinska Institutet, Stockholm, Sweden; Department of Oncology-Pathology, Laboratory of Translational Fertility Preservation, Karolinska Institutet, Stockholm, Sweden; Department of Medical Epidemiology and Biostatistics, Karolinska Institutet, Stockholm, Sweden; Uppsala Clinical Research Center, Uppsala University, Uppsala, Sweden; Department of Oncology-Pathology, Laboratory of Translational Fertility Preservation, Karolinska Institutet, Stockholm, Sweden; Department of Surgical Specialties, Biochemistry and Immunology, School of Medicine, University of Málaga, Málaga, Spain; IBIMA (Biomedical Research Institute of Málaga)-BIONAND Platform, University of Málaga, Andalucía Tech, Málaga, Spain; Department of Oncology-Pathology, Laboratory of Translational Fertility Preservation, Karolinska Institutet, Stockholm, Sweden; Department of Reproductive Medicine, Division of Gynecology and Reproduction, Karolinska University Hospital, Stockholm, Sweden

**Keywords:** frozen embryo transfer, fresh embryo transfer, crown–rump length, blastocyst transfer, cleavage-stage embryo transfer

## Abstract

**STUDY QUESTION:**

Is first-trimester intrauterine growth different between pregnancies resulting in live births established using fresh or frozen single embryo transfers (ET), and is it related to the embryo stage at the timing of transfer?

**SUMMARY ANSWER:**

Among pregnancies resulting in live birth, the use of frozen embryo transfer was associated with larger crown–rump length in the first trimester compared with fresh embryo transfer, regardless of the embryo stage at the timing of transfer.

**WHAT IS KNOWN ALREADY:**

Studies have indicated that singletons born following frozen/thawed ET have a higher likelihood of increased birthweight, in comparison with children born after fresh ET. A few studies have also suggested greater crown–rump length (CRL) in pregnancies after frozen embryo transfer (FET) compared to fresh ET. However, discrepancies exist regarding the intrauterine growth between fresh ET and FET groups, and the knowledge regarding the effect of embryo stage at transfer (cleavage stage vs. blastocyst stage) on the first-trimester growth remains limited.

**STUDY DESIGN, SIZE, DURATION:**

This prospective cohort study included all single ETs resulting in singleton pregnancies and live births following either FET or fresh ET at an academic reproductive medical center during 2013–2024.

**PARTICIPANTS/MATERIALS, SETTING, METHODS:**

Among 3445 singleton live births during the study period, 54.3% were after FET, and 62.2% after blastocyst transfer. All underwent an ultrasound scan between 6 and 12 gestational weeks to measure the CRL (mm). Generalized additive models were used to compare the CRL between pregnancies after FET and fresh ET, as well as to investigate associations according to embryo stage at transfer (cleavage stage or blastocyst stage), accounting for multiple confounders.

**MAIN RESULTS AND THE ROLE OF CHANCE:**

Compared with pregnancies after fresh ET, the CRL in pregnancies after FET was larger after adjusting for gestational age, embryo stage at transfer, parental age, maternal BMI, and smoking status in the first trimester, though the difference was small (β = 0.30 mm, 95% CI: 0.00–0.60, *P *= 0.053). The difference remained in subgroups defined by the embryo stage at the timing of transfer (β = 0.31 mm, 95% CI: −0.01–0.62, *P *= 0.055 and β = 0.22 mm, 95% CI: 0.06–0.39, *P *= 0.008 for cleavage and blastocyst stage, respectively). Normalizing CRL to a reference population conceived naturally from the INTERGROWTH-21(st) project showed that the mean CRL *Z*-scores in pregnancies after fresh ET and FET were both greater than 0 before day 63, suggesting the CRL in the present study population was larger than that of the non-ART population.

**LIMITATIONS, REASONS FOR CAUTION:**

The lack of adjustment for other relevant confounders, such as maternal infertility factors, could lead to unmeasured confounding. Additionally, the predominance of fresh embryos in the cleavage-stage ET group limited statistical power.

**WIDER IMPLICATIONS OF THE FINDINGS:**

A difference in the intrauterine growth of singletons born after frozen and fresh ETs appears early. The effect of FET on the CRL is similar regardless of whether cleavage-stage embryo or blastocyst transfer is used. Thus, the selection of embryo stage at the timing of transfer needs to be informed by other clinical factors. Besides, reliance on CRL for redating pregnancies conceived by assisted reproductive technologies warrants caution as it may overestimate the gestational age and miss potential growth restriction.

**STUDY FUNDING/COMPETING INTEREST(S):**

This study was supported by the Swedish Childhood Cancer Fund, Swedish Cancer Society, Radiumhemmets Research Funds, The Swedish Research Council, ALF Grants from Region Stockholm, and Karolinska Institutet research grants to KARW. ARP is supported by fundings of the Beatriz Galindo Program BG23/00015 with further support from FEDER and UE (PID2024-160756OA-I00) both from the Spanish Ministry of Science, Innovation and Universities and KI Research Foundation Grants 2024-2025(2024-02566). The funders had no role in the design and conduct of the study; collection, management, analysis, and interpretation of the data; preparation, review, or approval of the manuscript; and decision to submit the manuscript for publication. The authors declare no conflicts of interest regarding this work.

**TRIAL REGISTRATION NUMBER:**

N/A

WHAT DOES THIS MEAN FOR PATIENTS? Embryo culture and transfer are critical procedures in assisted reproductive treatments. This study looked at live births that were established using either fresh or frozen embryo transfer. The objective of the study was to investigate whether the first-trimester growth differs between those two groups and if it is related to the embryo stage at the timing of transfer (cleavage stage vs. blastocyst stage). We investigated 3445 singleton live-birth pregnancies and found that pregnancies after frozen embryo transfer were more likely to have a larger crown–rump length in the first trimester compared with those resulting from fresh embryo transfer, regardless of the embryo stage at the timing of transfer. This suggests that the choice of embryo stage at the timing of transfer needs to be informed by other clinical factors. Further research is warranted to support personalized treatment.

## Introduction

ART plays a central role in the field of fertility treatment. To date, more than 10 million children have been conceived by ART (Adamson *et al.*, 2025). Among ART methods, the use of frozen embryo transfer (FET) has notably increased in the last decade, accompanied by proposals for a ‘freeze-all’ strategy (Roque, 2015). This trend is largely driven by proposed benefits such as facilitating single embryo transfer policy and reducing maternal complications (Devroey *et al.*, 2011; Practice Committee of Society for Assisted Reproductive Technology and Practice Committee of American Society for Reproductive Medicine, 2012; Roque *et al.*, 2013; Alteri *et al.*, 2024). In addition, compared to fresh embryo transfer (ET), pregnancies after FET have been associated with better perinatal outcomes, including a lower risk of preterm birth and being small for gestational age. However, elevated risks of large for gestational age (LGA) and macrosomia have also been reported in pregnancies after FET (Sazonova *et al.*, 2012; Maheshwari *et al.*, 2018; Roque *et al.*, 2019; Terho *et al.*, 2021; Cavoretto *et al.*, 2022).

While many studies have focused on comparing the birthweight of babies born after FET versus fresh ET (Berntsen and Pinborg, 2018; Cavoretto *et al.*, 2022), only a few studies have investigated the intrauterine growth after FET and fresh ET, especially during early pregnancy when differences between the two groups might first become apparent (von Versen-Höynck *et al.*, 2018; Cavoretto *et al.*, 2021; Ageheim *et al.*, 2024; Mitta *et al.*, 2024; Yang *et al.*, 2024). Crown–rump length (CRL), a parameter widely used to assess the gestational age in the first trimester, is an important indicator of intrauterine growth. Recent studies have reported greater CRL in pregnancies after FET compared to fresh ET (Cavoretto *et al.*, 2021; Yang *et al.*, 2024). However, the timing of these differences varies across studies, partly due to variations in measurement timepoints and limited sample sizes. As a result, the precise timing and clinical significance of the intrauterine growth difference between FET and fresh ET remains unclear.

Parallel to the rise of FET, extended embryo culture to the blastocyst stage has become more prevalent in clinical practice. It was reported that blastocyst-stage transfer is associated with a higher cumulative live birth rate compared to cleavage stage (Saket *et al.*, 2021). However, emerging evidence shows that extending the embryo culture period increases the risk of LGA among infants born after FET, suggesting its potential effect on intrauterine growth (Pier *et al.*, 2024). In previous studies exploring the effect of FET on the CRL, the role of embryo stage at the timing of transfer was not considered. Thus, the aim of this study was to investigate whether intrauterine growth in the first trimester differs between pregnancies resulting in live births after FET and fresh ET, and to explore whether the stage of embryo transfer influences any observed differences.

## Materials and methods

### Ethical approval

The study was approved by the Regional Ethics Committee of Stockholm in 2011 (Dnr. 2011/1758-31/2) to allow prospective investigation of clinical outcomes of assisted reproduction and exempted specific signed consent for the study.

### Study participants

**Figure 1.**
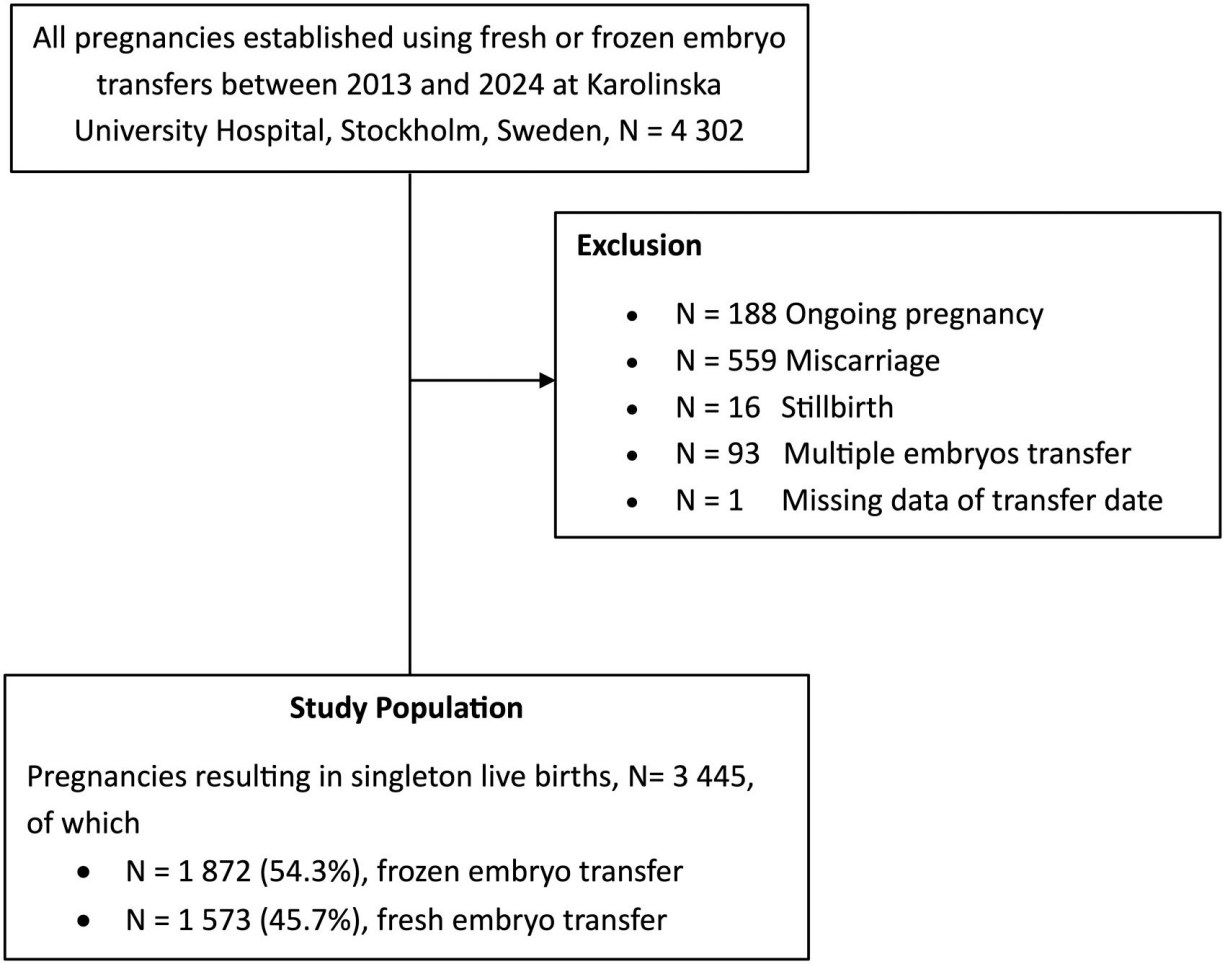
**Study flowchart.** Flowchart resulting in the final study population in the analysis.

This prospective study was conducted at Karolinska University Hospital between January 2013 and January 2024. Only singleton pregnancies resulting in live birth after single embryo transfers, either fresh or FET, were included. Of the 4302 IVF pregnancies during the study period, 575 were excluded due to miscarriage or stillbirth, and 282 were excluded because of multiple embryo transfer, unavailable pregnancy outcome, or missing data. Following exclusions, 3445 singleton pregnancies that resulted in live birth were included for analysis (Fig. 1). Information on ET type (frozen or fresh) and the embryo stage at the timing of transfer (cleavage-stage or blastocyst-stage) were retrieved from the local clinical registry, along with maternal characteristics such as age, BMI, smoking status in the first trimester, the results of early ultrasound exams to confirm a clinical pregnancy and the pregnancy outcome, which is reported to the center by the patients and double checked by a midwife accessing the regional obstetrics registry. In this study, all transfers were single embryo transfer (SET), and all frozen embryos were transferred on the same day of the thawing.

### CRL measurement

An ultrasound scan was performed regularly after ET to measure the first-trimester growth via CRL. In this study, CRL was measured as the greatest straight-line length from the cranial to the caudal end of the fetus in its natural supine position, between 6 and 12 gestational weeks (Robinson and Fleming, 1975).

### Statistical analyses

Gestational age at the time of the ultrasound scan was calculated to account for its influence on the CRL. In this study, it was determined by adding 14 days to the total of *in-vitro* embryo culture duration and the number of days between embryo transfer and the ultrasound scan. Considering the intrauterine growth pattern, CRL by gestational age was modeled using a generalized additive model (GAM), and the resulting smooth curves were plotted separately for the FET and fresh ET groups. GAM is an extension of linear regression, which allows for a possible non-linear relationship between the independent and dependent variables (Hastie and Tibshirani, 1995). For baseline characteristics between the FET and fresh ET groups, categorical variables were presented as frequencies and percentages and compared with the Chi-square (χ^2^) test, while continuous variables were summarized as medians with interquartile ranges and compared with the Mann–Whitney *U* test. To investigate the independent effect of FET on CRL, multiple factors including gestational age, the embryo stage at the timing of transfer, parental age, maternal BMI, and smoking status in the first trimester, were adjusted for in the models. The analyses were further stratified by embryo stage at transfer to examine whether the effect of FET on CRL varies with the timing of transfer. Additionally, CRL *Z*-scores were calculated to normalize CRL to a reference population in the INTERGROWTH-21(st) project in which an international prescriptive standard for intrauterine growth in the first trimester of pregnancy was produced (Papageorghiou *et al.*, 2014). The data were prepared and analyzed using SAS software (version 9.4, SAS Institute, Cary, NC, USA). Statistical significance was evaluated at an α-level of 0.05.

## Results

### Baseline characteristics

A total of 3445 pregnancies were included during the study period, and their baseline characteristics are shown in Table 1. Among these, 1872 (54.3%) pregnancies resulted from FET, of which 1780 (95.1%) involved blastocyst stage transfers. In contrast, 20.9% (329/1573) of the pregnancies from fresh ET were blastocyst transfers. The mean gestational age at the time of the ultrasound scan was the same in the FET and fresh ET groups (56 days). Baseline characteristics between the two groups were also similar, except for the parental age. Characteristics in the two subgroups categorized by the embryo stage at transfer (cleavage or blastocyst) are also shown in Table 1. Even though there is collinearity between embryo transfer type and the stage at transfer, the baseline characteristics between FET and fresh ET groups in each subgroup are still comparable, except for finding slightly higher paternal age in the FET group across both cleavage-stage embryos and blastocysts.

**Table 1.** Baseline characteristics of singleton pregnancies that resulted in live births following single embryo transfers of either fresh or frozen embryos between 2013 and 2024 at Karolinska University Hospital, Stockholm, Sweden.

	**Total pregnancies**		Pregnancies after cleavage-stage embryo transfer		Pregnancies after blastocyst transfer	
Characteristics	Frozen embryo transfer (n = 1872)	Fresh embryo transfer (n = 1573)	Frozen embryo transfer (n = 92)	Fresh embryo transfer (n = 1192)	Frozen embryo transfer (n = 1780)	Fresh embryo transfer (n = 329)
Gestational age at ultrasound test (days), median (IQR)	56.0 (54.0, 59.0)	56.0 (55.0, 58.0)	57.0 (55.0, 58.0)	56.0 (55.0, 58.0)	56.0 (54.0, 59.0)	55.0 (55.0, 59.0)
Embryo transfer stage, n (%)						
Cleavage stage	92 (4.9)	1192 (75.8)	92 (100.0)	1192 (100.0)	N/A	N/A
Blastocyst stage	1780 (95.1)	329 (20.9)	N/A	N/A	1780 (100.0)	329 (100.0)
Missing	0	52 (3.3)	N/A	N/A	N/A	N/A
Gender, n (%)						
Boy	959 (51.2)	582 (37.0)	37 (40.2)	435 (36.5)	922 (51.8)	129 (53.3)
Missing	76 (4.1)	443 (28.2)	24 (26.1)	341 (28.6)	52 (2.9)	87 (26.4)
Maternal age (years), median (IQR)	33.0 (30.0, 36.0)	33.0 (31.0, 36.0)	33.0 (31.0, 36.0)	34.0 (31.0, 36.0)	33.0 (30.0, 36.0)	33.0 (30.0, 36.0)
Maternal BMI (kg/m^2^)						
<18.5	39 (2.1)	24 (1.5)	4 (4.3)	15 (1.3)	35 (2.0)	9 (2.7)
18.5–29.9	1613 (86.2)	1330 (84.6)	75 (81.5)	1011 (84.8)	1538 (86.4)	277 (84.2)
>29.9	183 (9.8)	184 (11.7)	10 (10.9)	137 (11.5)	173 (9.7)	40 (12.2)
Missing	37 (2.0)	35 (2.2)	3 (3.3)	29 (2.4)	34 (1.9)	3 (0.9)
Maternal smoking, n (%)						
Yes	74 (4.0)	86 (5.5)	2 (2.2)	70 (5.9)	72 (4.0)	12 (3.6)
Missing	399 (21.3)	306 (19.5)	16 (17.4)	228 (19.1)	383 (21.5)	77 (23.4)
Paternal age (years)						
Median (IQR)	36.0 (32.0, 40.0)	35.0 (32.0, 39.0)	37.0 (34.0, 40.0)	35.0 (32.0, 39.0)	36.0 (32.0, 40.0)	34.0 (31.0, 39.0)
Missing	58 (3.1)	53 (3.4)	3 (3.3)	34 (2.9)	55 (3.1)	19 (5.8)

IQR: inter-quartile range.

### Difference in CRL between FET and fresh ET

**Figure 2.**
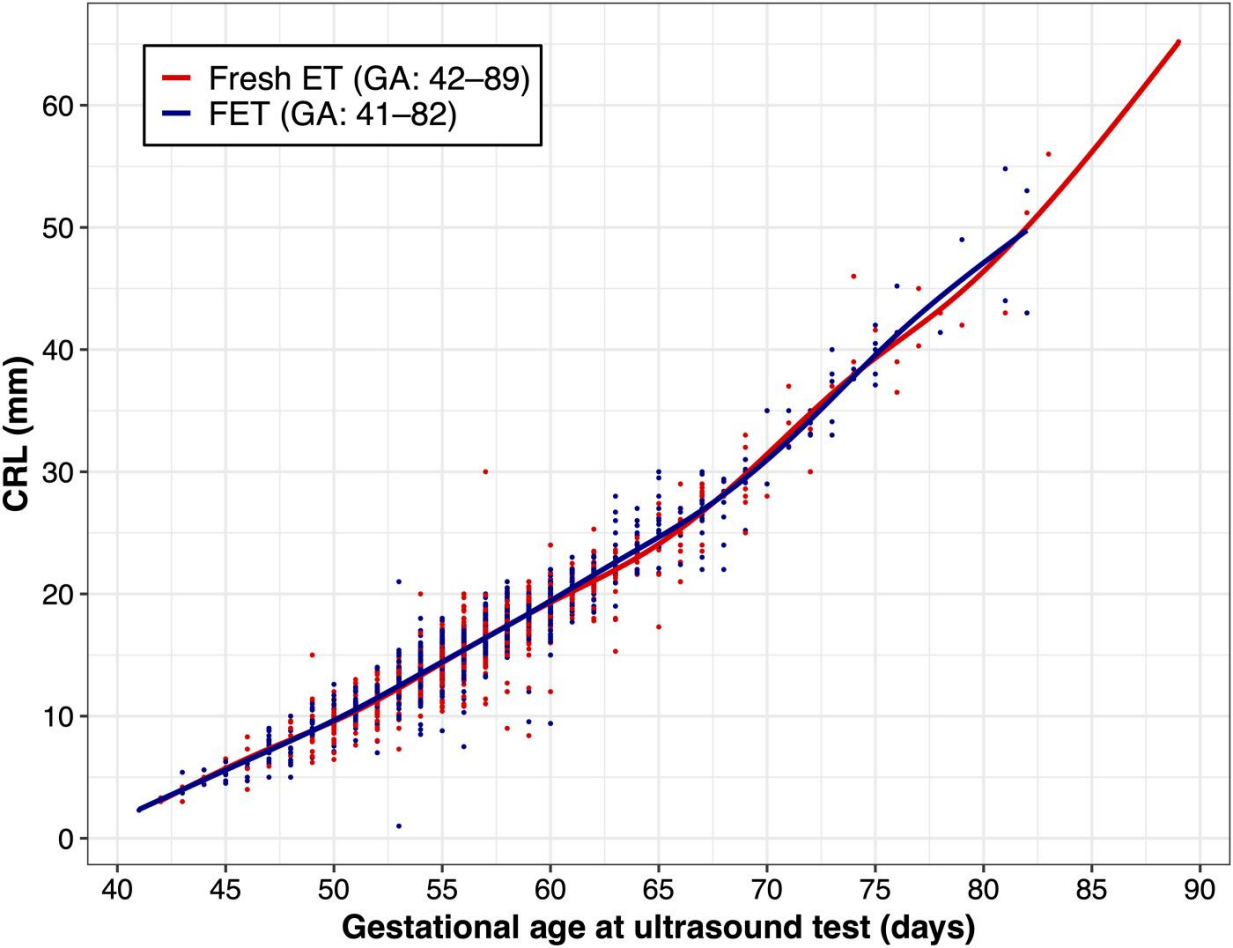
**The crown–rump length in pregnancies after frozen and fresh embryo transfer.** Distribution of crown–rump length (CRL) by gestational age in pregnancies following fresh (red) and frozen (blue) embryo transfer. Smooth lines represent predicted non-linear growth trajectories derived from generalized additive models for each group. CRL, crown-rump length; FET, frozen embryo transfer; ET, embryo transfer; GA, gestational age (in days).

The median CRL in fetuses after FET and fresh ET were 15.7 mm and 15.5 mm, respectively. The distribution of CRL based on gestational age in the two groups is shown in Fig. 2. The smooth line derived from the GAM demonstrates the non-linear growth pattern of CRL in the first trimester. Adjusting for gestational age, CRLs were similar between pregnancies after FET and fresh ET (β = 0.09, 95% CI: −0.01–0.19, *P *= 0.067). However, after also adjusting for the embryo stage at transfer, parental age, maternal BMI, and smoking status in the first trimester, FET was associated with a slightly greater mean CRL (β = 0.30, 95% CI: 0.00–0.60, *P *= 0.053).

To examine whether the association between FET and CRL differed by the embryo stage at the timing of transfer (cleavage-stage versus blastocyst-stage), subgroup analysis was conducted. In pregnancies after cleavage-stage ET, the median CRL in the FET group was larger than that of the fresh ET group (16.2 vs. 15.6 mm). After adjusting for confounders, the previously noted association remained (β = 0.31, 95% CI: −0.01–0.62, *P *= 0.055). Among pregnancies after blastocyst transfer, a larger median CRL in the FET group was also observed, compared to the fresh ET group (15.6 mm vs. 15.0 mm). The use of FET was also associated with larger mean CRL after adjusting for available confounders (β = 0.22, 95% CI: 0.06–0.39, *P *= 0.008).

### CRL in pregnancies after cleavage-stage and blastocyst-stage ET

The CRL in pregnancies after different transfer stages were compared (Supplementary Fig. S1). After adjusting for confounders, the mean CRL in pregnancies after cleavage-stage ET was slightly larger than in those transferred at blastocyst stage (β = 0.17, 95% CI: −0.01–0.34, *P *= 0.060). The difference between cleavage-stage and blastocyst-stage ET groups was greater in the FET group (β = 0.25, 95% CI: −0.04–0.54, *P *= 0.093) than that in the fresh ET group (β = 0.16, 95% CI: −0.02–0.34, *P *= 0.080), although the confidence intervals were largely overlapping.

### CRL *Z*-score

The mean CRL *Z*-scores in the FET and fresh ET group were 1.09 ± 0.92 and 1.06 ± 0.80 mm, respectively. The smooth line derived from the individual CRL *Z*-scores (Supplementary Fig. S2) showed that the mean CRL *Z*-scores in the FET and fresh ET groups were both larger than 0 before Day 63, suggesting a greater CRL compared to the reference population at the same gestational age.

## Discussion

In this prospective cohort study, compared to fresh ET, pregnancies following FET were associated with larger CRL in the first trimester, after adjusting for potential confounders. The CRL difference between the FET and fresh ET group remained regardless of whether pregnancies followed cleavage-stage or blastocyst transfers, suggesting the effect of FET on first-trimester growth is independent of embryo stage at the timing of transfer.

Several studies have investigated the early-stage intrauterine growth in pregnancies after FET and fresh ET. A growth difference between pregnancies after FET and fresh ET was first reported by Von Versen-Höynck *et al.* (2018) in a small cohort study where the CRL at 6–8 weeks of gestation was measured in 301 singleton pregnancies. Larger CRL was detected in pregnancies after FET, but the difference was small, similar to the difference found in the present study. The CRL difference in the first trimester was subsequently confirmed in three studies with larger sample sizes (Cavoretto *et al.*, 2021; Mitta *et al.*, 2024; Yang *et al.*, 2024). A recent longitudinal population-based cohort study reported larger CRL following FET as well, but only before 40 days’ gestation (Ageheim *et al.*, 2024). Although these studies yielded similar conclusions, the stage of ET—a critical aspect of ART—was not considered. In the past decade, there has been a steady shift in practice from the transfer of cleavage-stage embryos to blastocysts, especially for frozen embryos. In Sweden, the proportion of pregnancies after blastocyst transfer increased rapidly between 2007 and 2017, from 5% to 31% in fresh embryos and from 6% to 88% in frozen embryos (Saket *et al.*, 2021). Interestingly, singletons born after blastocyst transfer have been reported to have, on average, greater birthweight compared with singletons from cleavage-stage ET (Zhu *et al.*, 2014). In a recent large cohort study, blastocyst transfer was found to be an independent risk factor for LGA in pregnancies after FET, suggesting a role of ET stage on fetal development (Pier *et al.*, 2024).

To the best of our knowledge, the present study is the first to investigate the role of embryo stage in the effect of FET on intrauterine growth in early pregnancy. In line with the shift to more blastocyst transfer in clinical practice, 95.1% of the frozen embryos in this study were transferred at blastocyst stage compared to only 20.9% of the fresh ETs. Like previous findings, a larger CRL was found in pregnancies after FET compared with fresh ET in the first trimester. The result suggests an early growth difference at a group level, which also aligns with previous studies reporting higher birthweight and elevated risk of LGA in children born after FET compared with fresh ET. Furthermore, when the analysis was stratified by embryo stage, the greater CRL after FET remained, and the CRL difference between the FET and fresh ET groups was similar among those transferred at cleavage and blastocyst stage respectively. This indicates that the use of FET, rather than the embryo stage at transfer, is the primary factor associated with larger CRL in the first trimester, despite the high collinearity between FET and blastocyst transfer. This further suggests that the preference of embryo stage in FET should be considered from other clinical factors such as live birth rate or even long-term health.

Compared to the general population conceived spontaneously in the INTERGROWTH-21(st) project, the CRLs in this study population were larger before day 63, suggesting accelerated first-trimester growth in pregnancies after ART (either FET or fresh ET, or both). Larger CRL in pregnancies after ART were observed in most of the aforementioned studies and a recent Norwegian cohort study (Ageheim *et al.*, 2024; Mitta *et al.*, 2024; Seljeflot *et al.*, 2025), except for one in which fetuses conceived after ART had smaller CRL between 6 and 14 gestational weeks than the general population (Cavoretto *et al.*, 2021). Interestingly, accumulating evidence have reported opposite tendencies in pregnancies after FET and fresh ET regarding birthweight—FET is associated with an elevated risk of large for gestational age and macrosomia, while fresh ET is associated with an elevated risk of small for gestational age and low birthweight (Maas *et al.*, 2016; Spijkers *et al.*, 2017; Berntsen and Pinborg, 2018; Westvik-Johari *et al.*, 2021; Cavoretto *et al.*, 2022). Taken together, these findings suggest dynamic growth patterns between pregnancies after FET and fresh ET compared with spontaneous conception (SC). This is partially supported by an Italian study, which reported that pregnancies after FET and fresh ET both exhibited higher estimated fetal weight (EFW) than the SC group in the second trimester, but lower EFW in the late third trimester, with no divergence in intrauterine growth trajectories between FET and fresh ET groups (Cavoretto *et al.*, 2022). However, a recent population-based register study where EFW was also calculated during the pregnancy found that both FET and fresh ET groups started to have higher fetal weight than the SC group from gestational day 120, and the higher fetal weight in the FET group lasted until birth whereas the fresh ET group had lower fetal weight than the SC group from day 245 (Ageheim *et al.*, 2024). The differing results between these two studies may be attributed to different statistical models and study samples. Given the available evidence, reliance on CRL for redating pregnancies conceived by ART warrants caution, as it may overestimate the gestational age and miss potential growth restriction. Ultimately, more research is needed to investigate the longitudinal growth patterns throughout pregnancy following different embryo transfer types compared with SC.

Our findings support a positive association between FET and intrauterine growth in the first trimester. However, the CRL difference in pregnancies after FET and fresh ET was small, and whether it has any important clinical implications cannot be answered in the present study. While the underlying mechanism behind the growth difference remains unclear, there are several putative explanations. One is the effect of cryopreservation on the epigenetic reprogramming that occurs in preimplantation embryos (Reyes Palomares and Rodriguez-Wallberg, 2022; Wilkinson *et al.*, 2023). Growing evidence from animal models has shown that cryoprotective agents could cause epigenetic aberration in imprinted genes, a group of genes regulating fetoplacental development (Fowden *et al.*, 2006; Lambertini *et al.*, 2012; Sciorio *et al.*, 2024). A comparison of human placental tissues from pregnancies after FET and fresh ET has shown a higher methylation level on the *H19/IGF2* imprinting center, which would lead to lower *H19* expression and increased *IGF2* expression, following FET (Barberet *et al.*, 2021). This is in line with the greater first-trimester growth found in pregnancies after FET, as the silencing of *H19* and increased *IGF2* expression is proven to be associated with fetal overgrowth (Leighton *et al.*, 1995). Animal models have also shown that altered transcriptional changes introduced by embryo cryopreservation potentially explain the deviant early fetal growth following FET (Luo *et al.*, 2024). Another possible explanation for the observed growth difference could be the distinct intrauterine environment between pregnancies after FET and fresh ET. Ovarian stimulation is known to induce supraphysiological hormone levels in women. Consequently, embryos transferred in fresh ET cycles are exposed to an unphysiological environment, which has been found to be associated with a higher risk of low birthweight and small for gestational age (Imudia *et al.*, 2012; Pereira *et al.*, 2017). Furthermore, emerging evidence has reported a significantly higher first-trimester uterine artery pulsatility index (UtA-PI) in pregnancies after fresh ET compared with FET (Cavoretto *et al.*, 2020; Donno *et al.*, 2025). An increased UtA-PI, reflecting high blood flow resistance in the uterine arteries, is typically interpreted as impaired uteroplacental perfusion and has been linked with fetal growth restriction (Ferrazzi *et al.*, 2011). These findings collectively suggest that an altered intrauterine environment following fresh ET may contribute to the relatively smaller CRL observed in pregnancies after fresh ET. Beyond, several reports of higher birthweights in programmed FET compared with natural frozen cycles could further point to the corpus luteum playing a role in fetal growth (Ginström Ernstad *et al.*, 2019; Asserhøj *et al.*, 2021). However, data on endometrial preparation for FET are still contradictory. In our study population, FETs were preferably planned in natural cycles and HRT cycles only served as the last alternative option. Additional factors have been recognized as associated with FET negative outcomes, such as the use of double embryo transfer (DET) versus SET. In a large observational study including data of 11 612 FETs with known endometrial preparation in Sweden, the risks found were confined to DET, and when FETs categorized according to endometrial preparation were analyzed, the type of endometrial preparation did not alter the results (Rodriguez-Wallberg *et al.*, 2023). In this present study, only SETs resulting in singleton births were considered, limiting our research findings to this population. More human studies are warranted to explore the effect of cryopreservation and maternal hormone environment on intrauterine growth and their underlying mechanism.

Strengths of this study include the large sample size and relatively complete information on potential confounders. Several factors which are known to be associated with intrauterine growth in early pregnancy, such as maternal BMI, age, and smoking status, were adjusted for in this study. Almost all the pregnancies were conceived after a clinical diagnosis of infertility, which partially addresses the confounding from underlying infertility, even though specific diagnoses were not available. Subgroup analysis by embryo stage allowed investigation of the independent effect of FET on intrauterine growth in the first trimester and partially addressed the potential influence of underlying infertility, as the participants in the subgroup are more likely to have a similar infertility condition. For example, a poor ovarian responder is more likely to have a cleavage-stage ET in clinical practice. Still, we cannot exclude some residual confounding, and the predominance of fresh embryos in pregnancies after cleavage-stage ET and frozen embryos in the blastocyst transfer group limited statistical power. These facts are mainly determined by the clinical routines at our center, as we plan for transfer of embryos at cleavage stage if less than five fertilized oocytes are available, otherwise the planning is for transfer of blastocysts, and we do not perform egg retrievals or ETs on weekends; thus, it is the timing of the egg retrieval (day of the week) that determines the planning of the transfer (Feichtinger *et al.*, 2017). Additionally, restricting the study population to live-birth pregnancies is a limitation and may introduce potential collider-conditioning bias, depending on differences in live-birth rate between pregnancies after FET and fresh ET. Other limitations include the possibility of unmeasured confounding by parity and maternal co-morbidities such as cardiovascular diseases.

In conclusion, this study demonstrated that among pregnancies resulting in live births, the use of FET is associated with larger CRL in the first trimester, and the association appears independent of the stage of embryo transfer. The preference for embryo stage at the timing of transfer should be guided by other relevant clinical outcomes.

## Supplementary Material

hoag014_Supplementary_Data

## Data Availability

The data underlying this article cannot be shared publicly due to ethical considerations relating to the privacy of individuals who participated in the study.
