# Supplementary material for: First trimester growth after fresh or frozen single embryo transfer: embryo cleavage vs blastocyst stages
Source: Hum Reprod Open. 2026 Feb 17;2026(2):hoag014. doi: 10.1093/hropen/hoag014 (PMC12981912; doi:10.1093/hropen/hoag014)
Supplement: hoag014_Supplementary_Data [file hoag014_supplementary_data.docx]

**Supplementary Figure S1**

**a. The CRLs in pregnancies after frozen or fresh single cleavage-stage embryo transfer**

**b. The CRLs in pregnancies after frozen or fresh single blastocyst transfer**

**Supplementary Figure S1. a:** Distribution of crown-rump length by gestational age in pregnancies following fresh (red) and frozen (blue) single cleavage-stage embryo transfer. Smooth lines represent predicted non-linear growth trajectories derived from generalized additive models for each group. **b:** Distribution of crown-rump length by gestational age in pregnancies following fresh (red) and frozen (blue) single blastocyst transfer. Smooth lines represent predicted non-linear growth trajectories derived from generalized additive models for each group.

Abbreviation: CRL, crown-rump length; FET, frozen embryo transfer; ET, embryo transfer; GA, gestational age (in days)

**Supplementary Figure S2**

**The CRL *Z*-score in pregnancies after frozen or fresh single embryo transfer**

**Supplementary Figure S2.** Distribution of crown-rump length *Z*-score by gestational age in pregnancies following fresh (red) and frozen (blue) embryo transfer. Smooth lines represent predicted non-linear growth trajectories derived from generalized additive models for each group.

Abbreviation: CRL, crown-rump length; FET, frozen embryo transfer; ET, embryo transfer; GA, gestational age (in days)
